# Supplementary material for: Application of B cell immortalization for the isolation of antibodies and B cell clones from vaccine and infection settings
Source: Front Immunol. 2022 Dec 13;13:1087018. doi: 10.3389/fimmu.2022.1087018 (PMC9794141; doi:10.3389/fimmu.2022.1087018)
Supplement: Supplementary file 1 [file Presentation_1.pptx]

## Slide 1
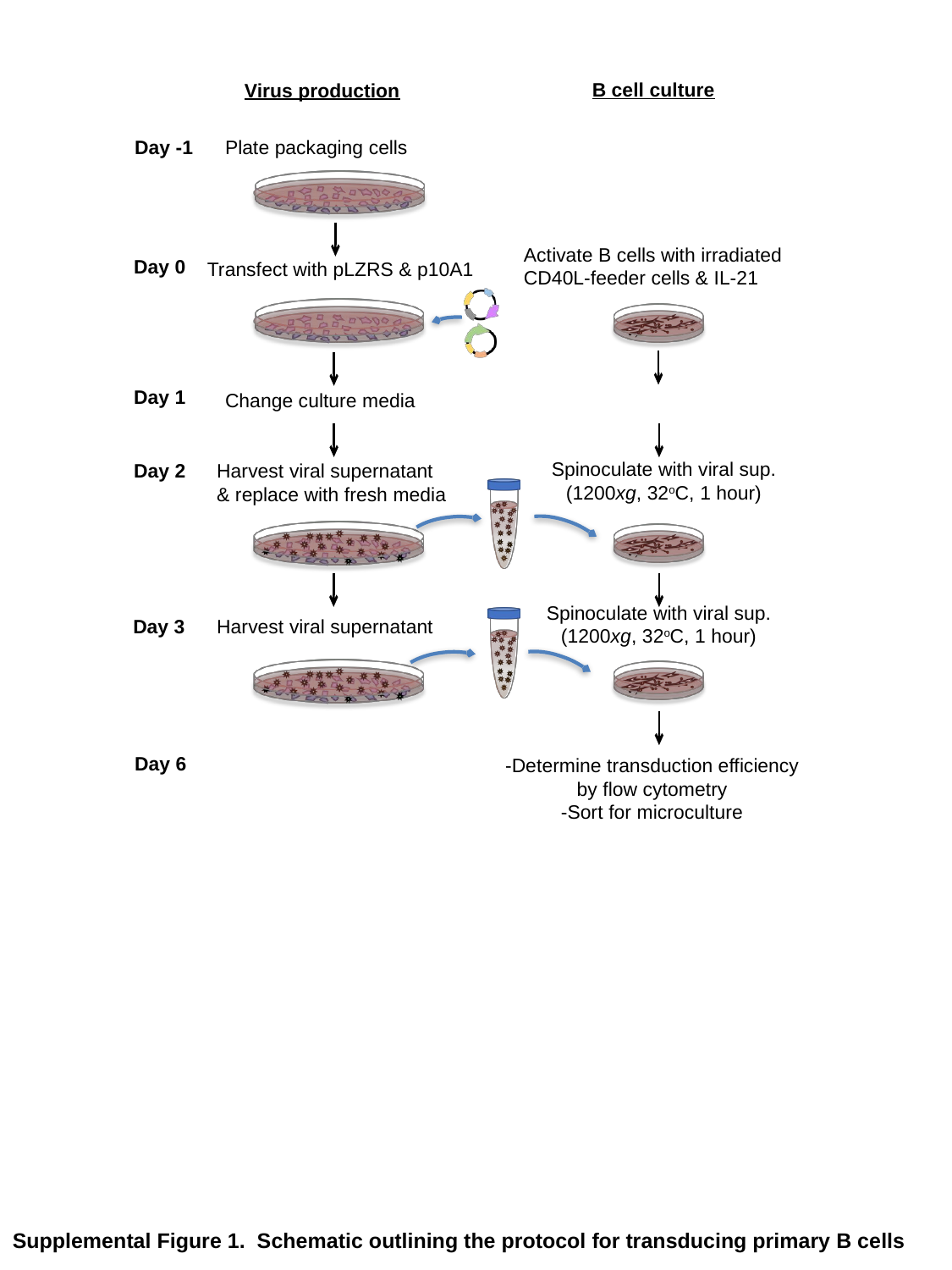

B cell culture
Virus production
Plate packaging cells
Day -1
Activate B cells with irradiated CD40L-feeder cells & IL-21
Day 0
Transfect with pLZRS & p10A1
Day 1
Change culture media
Spinoculate with viral sup.
(1200xg, 32oC, 1 hour)
Day 2
Harvest viral supernatant
& replace with fresh media
Spinoculate with viral sup.
(1200xg, 32oC, 1 hour)
Day 3
Harvest viral supernatant
Day 6
-Determine transduction efficiency by flow cytometry
-Sort for microculture
Supplemental Figure 1. Schematic outlining the protocol for transducing primary B cells

## Slide 2
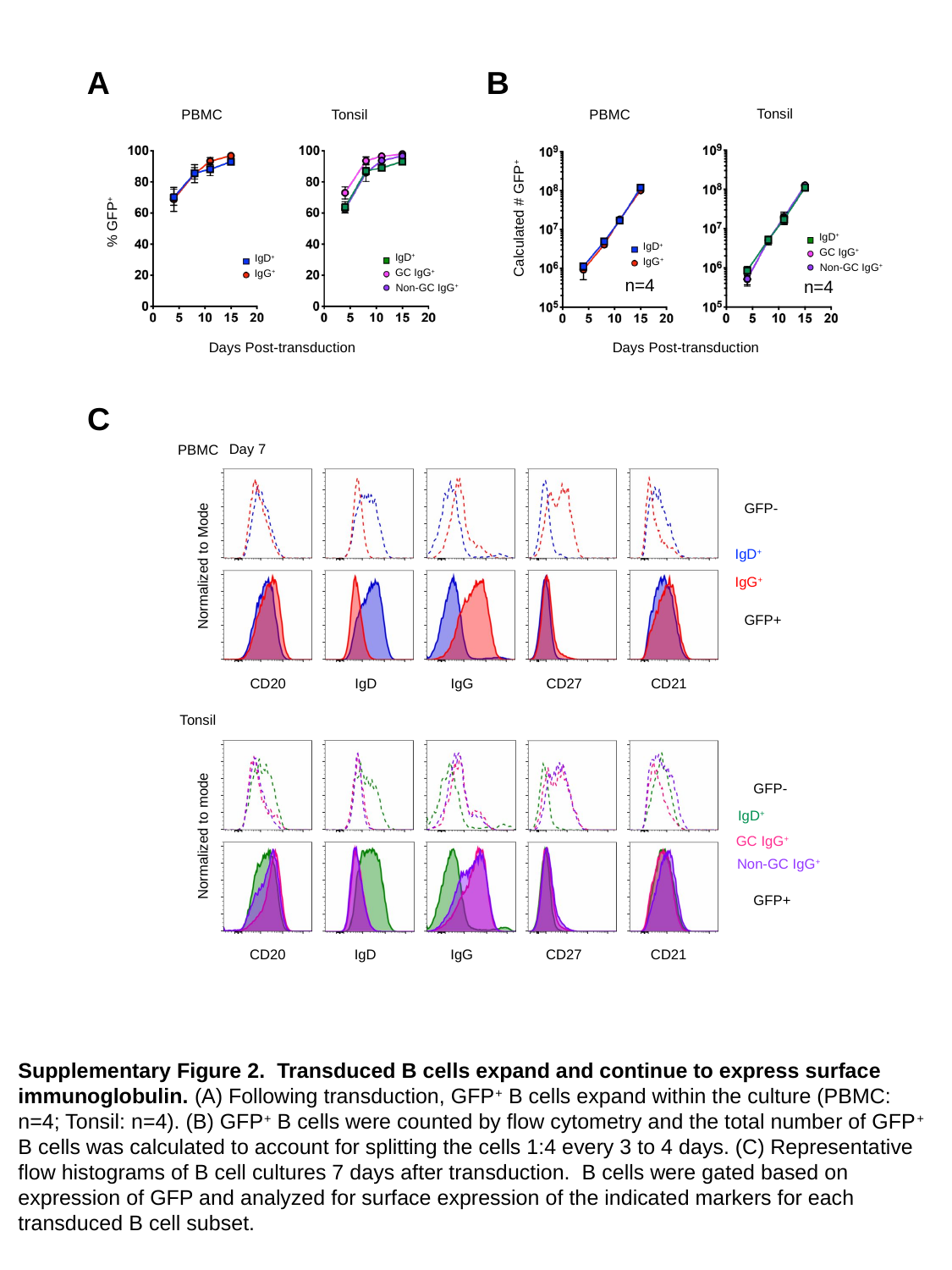

A
B
Tonsil
PBMC
PBMC
Tonsil
Calculated # GFP+
% GFP+
IgD+
IgD+
GC IgG+
IgD+
IgD+
IgG+
Non-GC IgG+
GC IgG+
IgG+
n=4
n=4
Non-GC IgG+
Days Post-transduction
Days Post-transduction
C
Day 7
PBMC
GFP-
IgD+
Normalized to Mode
IgG+
GFP+
CD20
IgD
IgG
CD27
CD21
Tonsil
GFP-
IgD+
Normalized to mode
GC IgG+
Non-GC IgG+
GFP+
CD20
IgD
IgG
CD27
CD21
Supplementary Figure 2. Transduced B cells expand and continue to express surface immunoglobulin. (A) Following transduction, GFP+ B cells expand within the culture (PBMC: n=4; Tonsil: n=4). (B) GFP+ B cells were counted by flow cytometry and the total number of GFP+ B cells was calculated to account for splitting the cells 1:4 every 3 to 4 days. (C) Representative flow histograms of B cell cultures 7 days after transduction. B cells were gated based on expression of GFP and analyzed for surface expression of the indicated markers for each transduced B cell subset.

## Slide 3
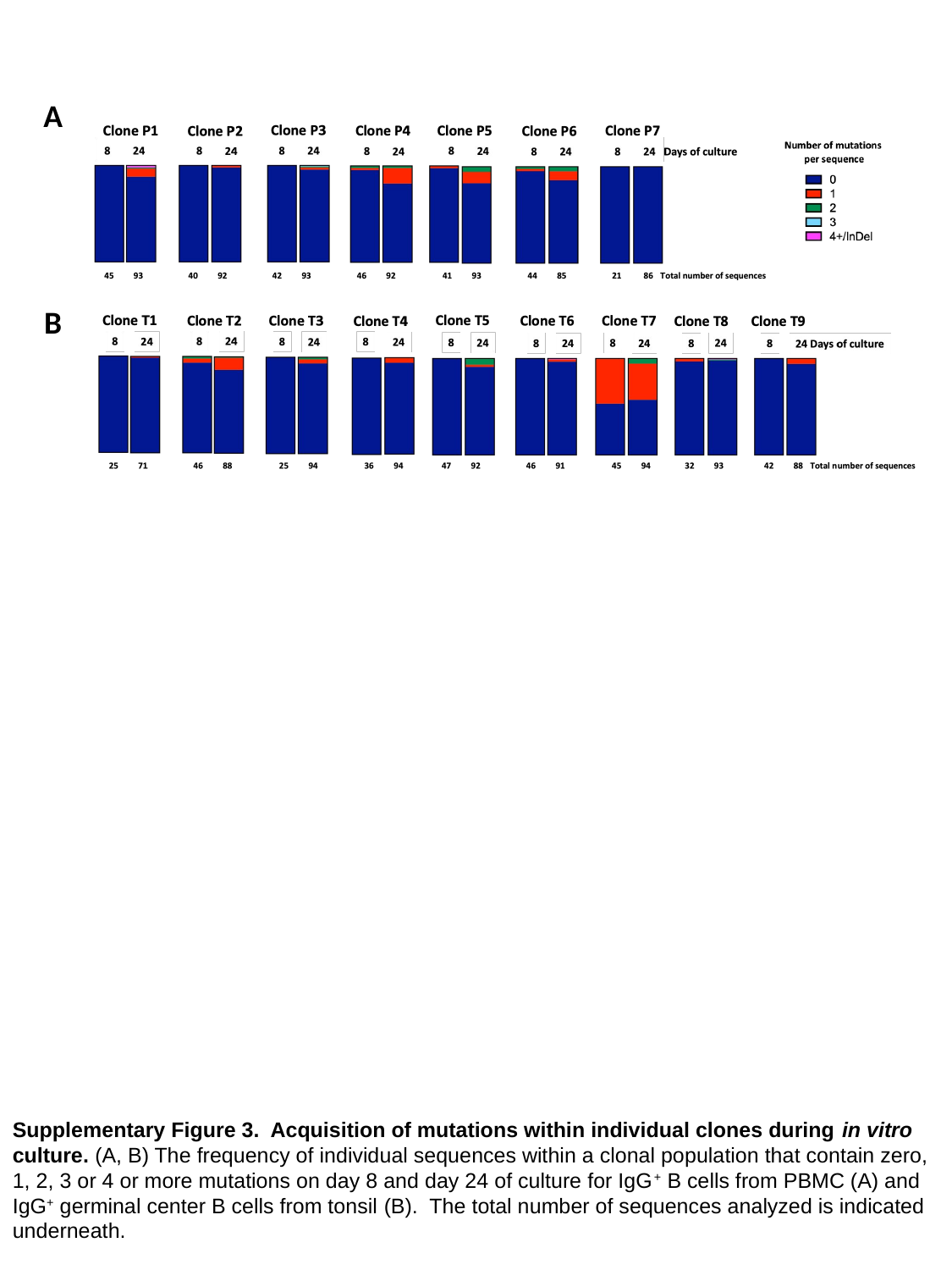

A
B
Supplementary Figure 3. Acquisition of mutations within individual clones during in vitro culture. (A, B) The frequency of individual sequences within a clonal population that contain zero, 1, 2, 3 or 4 or more mutations on day 8 and day 24 of culture for IgG+ B cells from PBMC (A) and IgG+ germinal center B cells from tonsil (B). The total number of sequences analyzed is indicated underneath.

## Slide 4
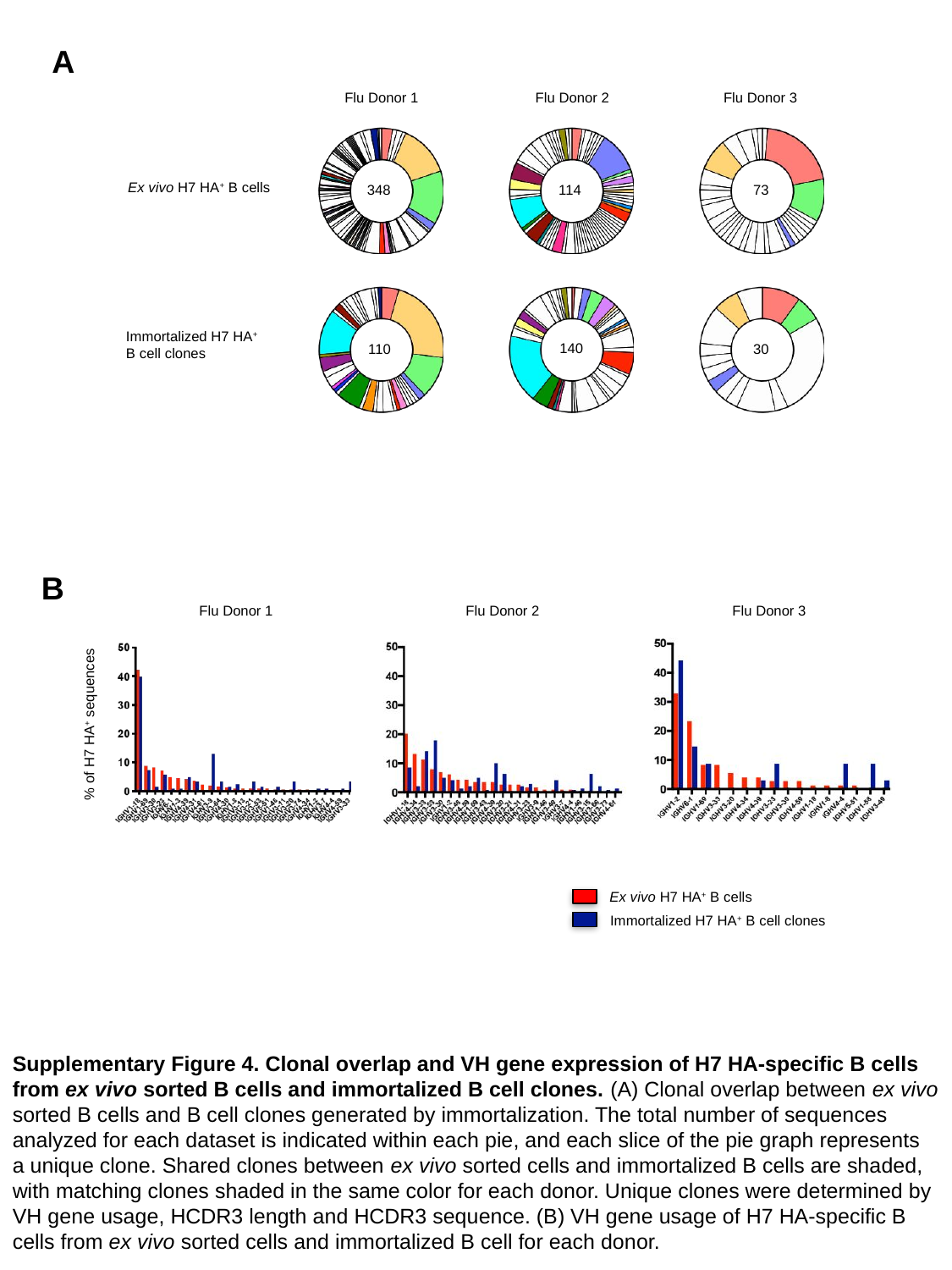

A
 Flu Donor 1
Flu Donor 2
Flu Donor 3
Ex vivo H7 HA+ B cells
348
114
73
Immortalized H7 HA+
B cell clones
140
30
110
B
 Flu Donor 1
Flu Donor 2
Flu Donor 3
% of H7 HA+ sequences
Ex vivo H7 HA+ B cells
Immortalized H7 HA+ B cell clones
Supplementary Figure 4. Clonal overlap and VH gene expression of H7 HA-specific B cells from ex vivo sorted B cells and immortalized B cell clones. (A) Clonal overlap between ex vivo sorted B cells and B cell clones generated by immortalization. The total number of sequences analyzed for each dataset is indicated within each pie, and each slice of the pie graph represents a unique clone. Shared clones between ex vivo sorted cells and immortalized B cells are shaded, with matching clones shaded in the same color for each donor. Unique clones were determined by VH gene usage, HCDR3 length and HCDR3 sequence. (B) VH gene usage of H7 HA-specific B cells from ex vivo sorted cells and immortalized B cell for each donor.

## Slide 5
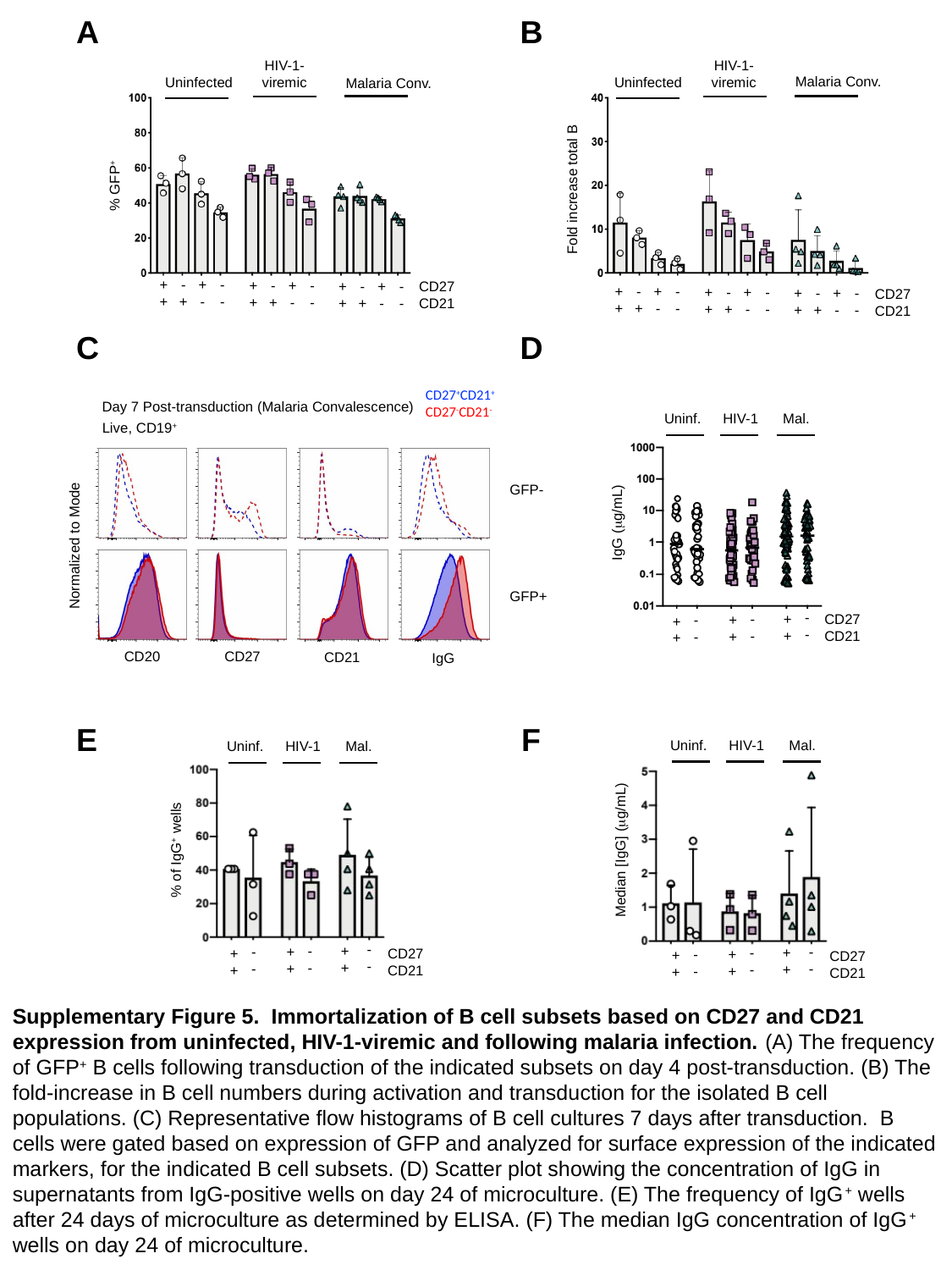

A
B
HIV-1-
viremic
HIV-1-
viremic
Malaria Conv.
Uninfected
Uninfected
Malaria Conv.
% GFP+
Fold increase total B
+
+
-
+
+
-
-
-
+
+
-
+
+
-
-
-
+
+
-
+
+
-
-
-
CD27
CD21
+
+
-
+
+
-
-
-
+
+
-
+
+
-
-
-
+
+
-
+
+
-
-
-
CD27
CD21
C
D
CD27+CD21+
CD27-CD21-
Day 7 Post-transduction (Malaria Convalescence)
Uninf.
HIV-1
Mal.
Live, CD19+
GFP-
IgG (mg/mL)
Normalized to Mode
GFP+
-
-
-
-
CD27
CD21
+
+
-
-
+
+
+
+
CD20
CD27
CD21
IgG
E
F
Uninf.
HIV-1
Mal.
Uninf.
HIV-1
Mal.
% of IgG+ wells
Median [IgG] (mg/mL)
-
-
-
-
+
+
-
-
-
-
+
+
-
-
+
+
+
+
CD27
CD21
-
-
+
+
+
+
CD27
CD21
Supplementary Figure 5. Immortalization of B cell subsets based on CD27 and CD21 expression from uninfected, HIV-1-viremic and following malaria infection. (A) The frequency of GFP+ B cells following transduction of the indicated subsets on day 4 post-transduction. (B) The fold-increase in B cell numbers during activation and transduction for the isolated B cell populations. (C) Representative flow histograms of B cell cultures 7 days after transduction. B cells were gated based on expression of GFP and analyzed for surface expression of the indicated markers, for the indicated B cell subsets. (D) Scatter plot showing the concentration of IgG in supernatants from IgG-positive wells on day 24 of microculture. (E) The frequency of IgG+ wells after 24 days of microculture as determined by ELISA. (F) The median IgG concentration of IgG+ wells on day 24 of microculture.

## Slide 6
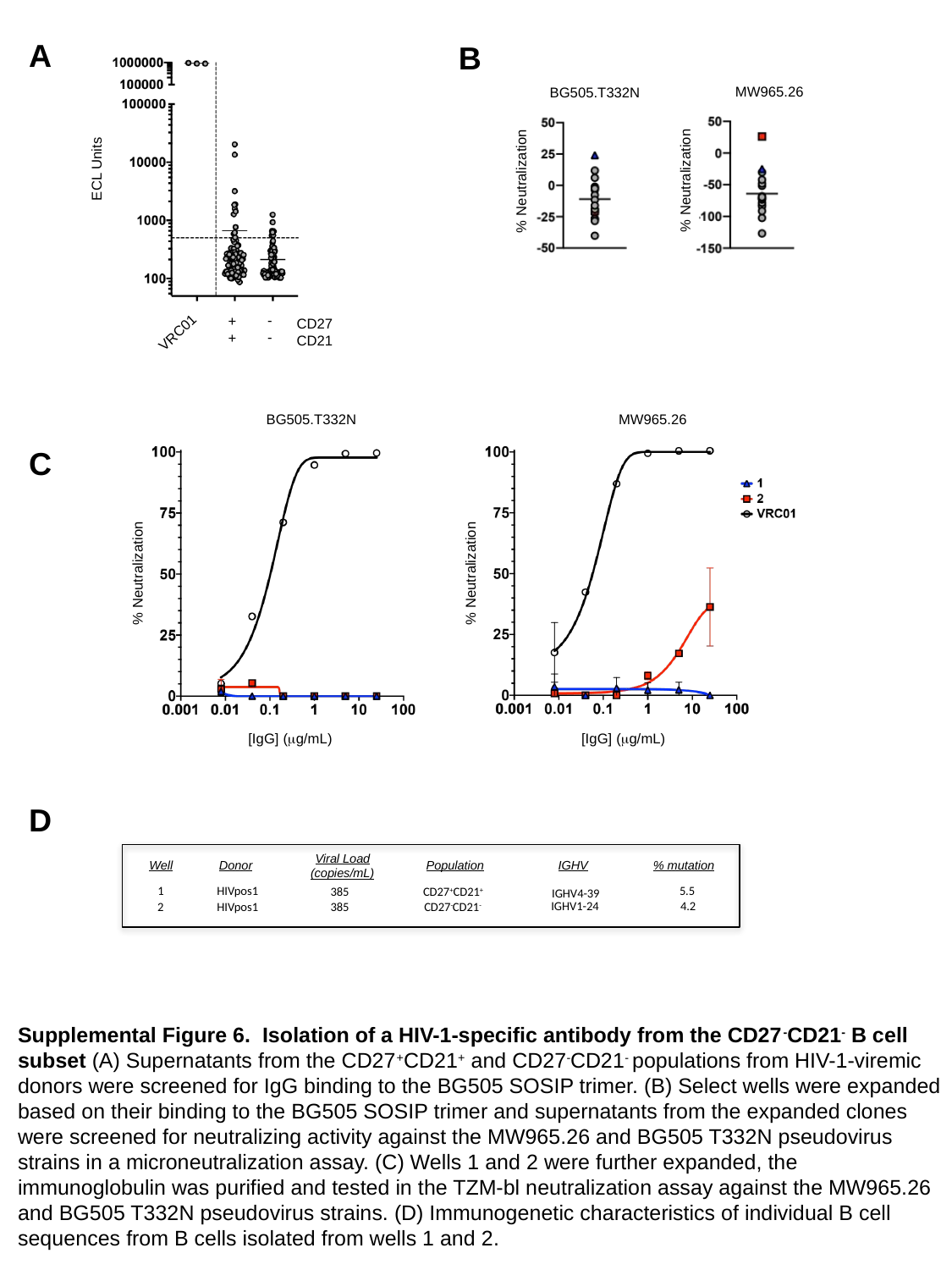

A
B
MW965.26
BG505.T332N
ECL Units
% Neutralization
% Neutralization
-
-
+
+
CD27
CD21
VRC01
BG505.T332N
MW965.26
C
% Neutralization
% Neutralization
[IgG] (mg/mL)
[IgG] (mg/mL)
D
Viral Load (copies/mL)
IGHV
% mutation
Population
Donor
Well
5.5
1
HIVpos1
385
CD27+CD21+
IGHV4-39
IGHV1-24
4.2
2
385
HIVpos1
CD27-CD21-
Supplemental Figure 6. Isolation of a HIV-1-specific antibody from the CD27-CD21- B cell subset (A) Supernatants from the CD27+CD21+ and CD27-CD21- populations from HIV-1-viremic donors were screened for IgG binding to the BG505 SOSIP trimer. (B) Select wells were expanded based on their binding to the BG505 SOSIP trimer and supernatants from the expanded clones were screened for neutralizing activity against the MW965.26 and BG505 T332N pseudovirus strains in a microneutralization assay. (C) Wells 1 and 2 were further expanded, the immunoglobulin was purified and tested in the TZM-bl neutralization assay against the MW965.26 and BG505 T332N pseudovirus strains. (D) Immunogenetic characteristics of individual B cell sequences from B cells isolated from wells 1 and 2.
